# Novel Active Films with Semolina and Jatoba (*Hymenaea courbaril* L.): Preparation, Properties, and Sustainability Aspects

**DOI:** 10.3390/foods14132391

**Published:** 2025-07-06

**Authors:** Cristiani Viegas Brandão Grisi, Flávia Cosmo Guedes da Silva, Rita de Cassia Andrade Silva, Rene Pinto da Silva, Fábio Anderson Pereira da Silva, Angela Maria Tribuzy de Magalhães Cordeiro

**Affiliations:** 1 Postgraduate Program in Agrifood Technology, Federal University of Paraiba, Campus Universitario III, Bananeiras 58220-000, PB, Brazil; flavia.cguedes@hotmail.com (F.C.G.d.S.); fabio.silva@academico.ufpb.br (F.A.P.d.S.); atribuzycordeiro@gmail.com (A.M.T.d.M.C.); 2 Postgraduate Program in Chemistry, Department of Chemistry, Federal University of Paraiba (UFPB), João Pessoa 58051-900, PB, Brazil; ritaandrade112@gmail.com; 3 Department of Food Technology, Center of Technology and Regional Development, Federal University of Paraiba, João Pessoa 58055-000, PB, Brazil; rene-qi@hotmail.com

**Keywords:** antioxidant packaging, active films, biodegradability, jatoba (*Hymenaea courbaril* L.), phenolic compounds

## Abstract

The aim of this study was to develop and characterize antioxidant-active films for potential food packaging applications. The films were produced by casting aqueous solutions containing semolina flour (6% *w*/*w*), pectin extracted from passion fruit (1% *w*/*w*), inverted sugar (1% *w*/*w*), and sucrose (1% *w*/*w*), incorporating hydroalcoholic extracts from jatoba stem bark (X_1_) and pods (X_2_) at concentrations ranging from 0 to 1% (*w*/*w*). The films were characterized in terms of their functional, physical, chemical, structural, and degradation properties. The formulation that showed the best performance, referred to as the optimized formulation (FO), contained 0.5% X_1_ and 0.5% X_2_, presenting a high phenolic compound content (8.80 mg GAE/g), strong antioxidant activity as determined by the DPPH method (75.28%) and FRAP assay (6.02 mmol FeSO_4_/g), good thermal stability (350 °C), and a high soil degradation rate (83.47% in 15 days). These results indicate that the FO film has potential application as a primary packaging material with antioxidant function for oxidation-sensitive foods, meeting the demand for biodegradable and environmentally sustainable solutions in the food industry.

## 1. Introduction

The growing concern over the environmental impact caused by the improper disposal of conventional packaging, particularly those made from synthetic polymers, has driven the search for more sustainable alternatives. The packaging industry has pursued the development of new materials, including biodegradable films, edible packaging, recyclable alternatives, and advanced technologies for active and intelligent packaging [1]. Synthetic plastics, owing to their high durability and resistance to degradation, can persist in the environment for hundreds or even thousands of years, exacerbating environmental pollution. The partial or complete replacement of these materials with bio-based alternatives has attracted increasing interest from both consumers and researchers, stimulating the search for environmentally friendly and resource-efficient raw materials [2].

Among the promising materials for the development of biodegradable packaging are biomolecules such as polysaccharides, lipids, and proteins, which can be degraded by microorganisms within a short period [3]. Polysaccharides are particularly attractive due to their biodegradability, biocompatibility, and abundance in various plant sources [4]. These sources include both raw plant materials, such as cassava, yam, taro, and potato, and processed derivatives, such as semolina derived from wheat. While cassava and yam are typically used in their more natural forms, semolina is a refined product. These materials have been widely applied in the production of active packaging films due to their compatibility with bioactive compound incorporation, which imparts additional functionalities, such as antioxidant or antimicrobial properties [5].

Active packaging represents an advancement over conventional packaging by interacting in a controlled manner with food and its surrounding environment, thereby contributing to the preservation of quality and extension of the product shelf life [6]. A central focus in the development of such packaging is the incorporation of plant-based bioactive compounds, known for their antioxidant and antimicrobial properties, which can be extracted from various parts of plants, such as peels, seeds, and pulp residues [7]. Studies on native plant species from biomes such as the Caatinga [8] and Cerrado [9] have demonstrated the potential of natural extracts for formulating active films. These bioactive compounds can neutralize the free radicals responsible for the oxidative degradation of food, thus contributing to the preservation of freshness and quality over time [10].

In this context, *Hymenaea courbaril* stands out as a native plant of the Cerrado biome with recognized social and economic importance. Its pods and bark from the stem have traditionally been used, for example, in teas and in the consumption of raw pulp [11]. However, the technological applications of jatobá remain underexplored. Studies have shown a high phenolic compound content in the pulp (15.7 mg/100 g) and significant antioxidant activity (44.30% inhibition using the DPPH method) [12]. These findings underscore the potential of jatoba as a source of bioactive compounds for active packaging applications.

Simultaneously, semolina, a byproduct of wheat processing, has emerged as a viable matrix for producing biodegradable films. According to Jafarzadeh et al. [13], semolina possesses suitable structural properties to serve as the foundation of a film-forming matrix, resulting in films with favorable physical and mechanical characteristics for sustainable applications.

In this context, the hypothesis of this study is that incorporating bioactive compounds, such as those found in jatoba, into the semolina matrix can enhance the functional properties of the films, promoting the development of active materials with promising applications in the food packaging industry. Therefore, this study aimed to optimize the conditions for producing semolina-based biodegradable films enriched with jatoba extract and evaluate their physical, mechanical, thermal, and antioxidant properties. This study aims to contribute to the advancement of innovative solutions in active packaging, with the potential to extend the shelf life of food products and reduce the environmental impact associated with the use of conventional plastic materials.

## 2. Materials and Methods

### 2.1. Materials

Bark from the stems and pods of jatoba (*Hymenaea courbaril*) was purchased from a local market in Patos, Paraíba, Brazil. Passion fruit, sugar, and semolina flour were obtained from a local market in João Pessoa, Paraíba, Brazil.

### 2.2. Preparation of Plant Extracts

The barks from the stem (X_1_) and pod (X_2_) of jatoba were dried at 40 °C in an air-circulating oven for 24 h. Subsequently, they were ground using a knife mill, and the resulting powder was used for extract preparation. For extraction, 10 g of powder was dissolved in 100 mL of a water/ethanol solution (30:70, *v*/*v*) at a ratio of 1:10 (*w*/*v*). The extraction time was 2 h at 40 °C with constant agitation in a shaking incubator (Technal, São Paulo, Brazil). Subsequently, vacuum filtration was performed, and the resulting extract was concentrated using a rotary evaporator for 10 min at 60 °C [14]. The concentrated extracts were stored in amber bottles, protected from light, and refrigerated at 5 °C until further analysis.

### 2.3. Extraction of Pectic Extract from Yellow Passion Fruit

Passion fruits were sanitized in a 100 ppm sodium hypochlorite solution, rinsed under running water, and then halved to extract the pulp and seeds. The peel (including the albedo) was cut into smaller pieces and dried. For extraction, 500 mL of distilled water, 200 g of peel, and 20 g of lemon juice were used. Initially, the water and albedo were heated until softening. Lemon juice was then added to the mixture and heated for an additional 10 min. The pectic extract from passion fruit was stored in a glass container at 5 °C [15].

### 2.4. Preparation of Inverted Sugar

The mixture containing sucrose and water (1:1, *w*/*w*) was manually homogenized and heated at 120 °C for 20 min to ensure its complete dissolution. Then, 3.6 mL of lemon juice (*Tahiti variety*) was added, and the solution was maintained at the same temperature for an additional 5 min. The resulting inverted sugar was transferred to a glass container, allowed to cool, and stored at room temperature (25 °C) [16].

### 2.5. Experimental Design and Film Preparation

The films were prepared according to the methodology described by Fernandes et al. [16] using the casting solution technique. The films were produced from an aqueous film-forming solution containing semolina flour (6% *w*/*w*), passion fruit pectin extract (1% *w*/*w*), inverted sugar (1% *w*/*w*), sucrose (1% *w*/*w*), and extracts from X_1_ and X_2_ (0–1% *w*/*w*). The aqueous solution was heated in a microwave until it reached a gelation temperature of 65–70 °C. A total of 45 g of the film-forming solution was dispensed into polystyrene Petri dishes (140 × 15 mm) and subsequently dried in a forced-air oven at 40 ± 2 °C for 12 h. The films were stored at 75% relative humidity and 25 °C for 10 days before characterization.

The conditions for film preparation were optimized using a central composite rotational design (CCRD), which consisted of a factorial design with four axial points and three central points, totaling 11 formulations. The independent variables were (X_1_) the content of stem bark extract and (X_2_) the content of pod extract from jatoba, both ranging from 0–1% (Table 1). The response variables were antioxidant activity (DPPH and FRAP) and total phenolic content (TPC).

The ranges of the two independent variables were determined based on the results of preliminary experiments. The dependent variables were total phenolic content (TPC) and antioxidant activity (DPPH and FRAP assays).

The levels of the two independent variables were determined through preliminary experiments. The total phenolic content (TPC) and antioxidant activity, assessed using DPPH and FRAP assays, were selected as the dependent variables. The response surface was constructed using a mathematical model represented by Equation (1), where Y denotes the response variables (TPC, DPPH, FRAP), β represents the model coefficients, X_1_ and X_2_ are the independent factors, and the random error.

Y = ± β_0_ ± β_1_X_1_ ± β_2_X_2_ ± β_12_X_1_X_2_ ± β_11_X_1_^2^ ± β_22_X_2_^2^ + e (1)

The TPC, DPPH, and FRAP values were measured under optimized conditions to validate the model. After film optimization, physical, mechanical, barrier, and thermal characterizations were performed. The optimized formulation was compared with a control film without the addition of the extracts (FC).

### 2.6. Film Characterization

#### 2.6.1. Mechanical Properties and Thickness

The tensile strength (TS) and elongation at break (EB) were determined using a SHIMADZU static testing instrument in accordance with ASTM D882-12 [17]. The film specimens were cut into rectangular strips (10 × 1.5 mm) with an initial grip separation of 50 mm and a crosshead speed of 10 mm/min. Six replicates were analyzed per treatment, and the results were expressed in megapascals (MPa) for TS and as a percentage (%) for EB. The film thickness was measured at room temperature (25 °C) using a digital caliper with a precision of 0.001 mm. Ten measurements were randomly taken approximately 60 mm from the edges, and the average value was used to calculate the mechanical properties, with the results reported in millimeters (mm).

#### 2.6.2. Color Analysis

The film color was evaluated using a GretagMacbeth—Coloreye 2180 colorimeter (GretagMacbeth, São Paulo, Brazil) with a D65 standard illuminant and a 10° standard observer [18]. Each sample was placed on a white reference background, and the color values were obtained using the CIELAB system. In this system, L* denotes brightness (ranging from 0 for black to 100 for white), while a* and b* indicate chromatic coordinates: a* spans from green (−) to red (+), and b* from blue (−) to yellow (+). For each type of film, five measurements were taken at random locations and averaged across three replicates. The color difference (ΔE) relative to the control sample (FC) was determined using Equation (2), as follows:

ΔE = [(ΔL*)^2^ +( Δa*)^2^ + (Δb*)^2^]^1/2^ (2)

where ΔL* = Lstandard − Lsample, Δa* = astandard − asample, and Δb* = bstandard − bsample.

#### 2.6.3. Water Solubility

Water solubility was evaluated as described by Ojagh et al. [19], with modifications. The film samples were cut into 2 × 2 cm squares, and their initial dry weights were obtained by drying at 60 °C for 24 h. The samples were then immersed in 50 mL of distilled water in Erlenmeyer flasks (250 mL) and agitated at 100 rpm (MultiShaker, São Paulo, Brazil) for 24 h at 25 °C. Following immersion, the films were dried again at 60 °C for 24 h to determine the mass of the undissolved dry matter. The water solubility was calculated using Equation (3).

(3) $$Solubility\;\left(\%\right)=(\frac{mi-mf}{mi})\times 100$$

#### 2.6.4. Water Vapor Permeability (WVP)

Water vapor permeability (WVP) tests were performed using the gravimetric method based on the ASTM E96/E96M-16 protocol [20] with the necessary adaptations. Films with a diameter of 5 cm were placed and sealed in acrylic containers containing silica gel. The film-container system was then placed in a desiccator (25 °C) at 75% relative humidity using saturated NaCl solution. The samples were weighed on an analytical balance every 24 h for 7 days in triplicate. WVP, expressed in gH_2_O·mm/m^2^·h·mmHg, was calculated using Equation (4):

(4) $$\;WVP=\left(\frac{∆m}{∆t}\right)\times \left(\frac{X}{Ps\;\cdot \;(RH1-RH2)}\right)$$

where WVP is the water vapor permeability, Δm/Δt is the slope of the weight gain curve per unit time, A is the exposed surface area of the film (m^2^), X is the average film thickness, Ps is the saturation pressure of the water, RH1 is the relative humidity inside the desiccator (75%), and RH2 is the relative humidity inside the capsule (0%).

#### 2.6.5. Total Phenolic Compound Content and Antioxidant Activity

To determine the total phenolic compound content and antioxidant activity using DPPH and FRAP, 100 mg of the film samples were homogenized with 10 mL of distilled water for 2 h. The samples were subsequently centrifuged, and the resulting supernatant was collected for further analysis.

Determination of Total Phenolic Content: The concentration of phenolic compounds was determined following the method described by Slinkard and Singleton [21]. For the assay, a 150 μL aliquot of the sample reacted with the Folin-Ciocalteu reagent. Absorbance was measured at 760 nm using a spectrophotometer. To quantify the phenolic content, a calibration curve was generated using gallic acid standards at concentrations between 1 and 20 mg/L, prepared under the same conditions as the samples. The results are expressed as milligrams of gallic acid equivalents per gram of the sample (mg GAE/g).

2,2-Diphenyl-1-picrylhydrazyl (DPPH●): The antioxidant capacity was assessed using the DPPH (2,2-diphenyl-1-picrylhydrazyl) assay, adapted from the method of Brand-Williams, Cuvelier, and Berset [22] with modifications. Samples (150 µL), diluted with an equal volume of ethanol, were mixed with 2.7 mL DPPH solution prepared in ethanol. The reaction mixture was incubated in the dark for 30 min, after which the absorbance was measured at 517 nm using a spectrophotometer (Quimis, model Q798U, Joinville, Brazil). A DPPH-only solution was used as the control. Antioxidant activity was expressed as the percentage of radical-scavenging inhibition, calculated using Equation (5).

(5) $$\%inhibition\;DPPH=\frac{ABScontrol-ABSsample}{ABScontrol}\times 100$$

where *ABScontrol* is the control reaction absorbance, and *ABSsample* is the sample absorbance.

Capacity by the ferric reducing antioxidant power (FRAP) method: Antioxidant activity was evaluated using the FRAP assay adapted from the method described by Rufino et al. [23]. The FRAP reagent was freshly prepared by mixing acetate buffer (300 mM, pH 3.6), TPTZ (2,4,6-tripyridyl-s-triazine) solution (10 mmol/L in 40 mmol/L HCl), and FeCl_3_ solution (20 mmol/L) in a 10:1:1 (*v*/*v*/*v*) ratio. The sample (90 μL) was combined with 270 μL of distilled water and 2.7 mL of the FRAP reagent in test tubes. The mixtures were homogenized and incubated at 37 °C for 30 min in a water bath. Absorbance was measured at 595 nm using a spectrophotometer, with the FRAP reagent serving as the blank. A standard curve was generated using FeSO_4_ solutions ranging from 50 to 2000 μM. The results were expressed as millimoles of FeSO_4_ equivalents per gram of sample (mmol FeSO_4_/g).

#### 2.6.6. Thermogravimetric Analysis (TG/DTG)

The thermal stabilities of the films were evaluated using a SHIMADZU DTG-60H thermal analyzer (SHIMADZU, Barueri, Brazil). Approximately 5–10 mg of each sample was weighed into an alumina crucible and heated under a nitrogen atmosphere at a flow rate of 50 mL/min. The temperature was increased at a constant rate of 10 °C/min, covering the range from 30 to 550 °C [24]. Thermograms showing the percentage of mass loss as a function of temperature were generated using the OriginPro software (version 8.5; OriginLab Corporation, Northampton, MA, USA ).

#### 2.6.7. Differential Scanning Calorimetry (DSC)

The thermal transitions of the films were analyzed using a DSC-60 calorimeter (SHIMADZU, Barueri, Brazil) under a nitrogen atmosphere at a flow rate of 20 mL/min. Samples weighing 5–10 mg were sealed in alumina crucibles prior to analysis. The temperature program ranged from 30 to 550 °C at a heating rate of 10 °C/min [24]. The heat flow corresponding to the thermal transitions of the film components was recorded and processed using OriginPro software (version 8.5; OriginLab Corporation, Northampton, MA, USA).

#### 2.6.8. X-Ray Diffraction (XRD)

The crystalline structures of the films were examined using the method described by Spagnol et al. [25]. XRD measurements were performed using a Shimadzu XRD-6000 diffractometer (Tokyo, Japan) operating at 40 kV and 30 mA with CuKα radiation (λ = 1.5418 Å). The analysis was conducted over a 2θ range of 10–50° at a scanning rate of 2°/min. The obtained diffraction patterns were analyzed and interpreted using the OriginPro software (version 8.5; OriginLab Corporation, Northampton, MA, USA).

#### 2.6.9. Fourier-Transform Infrared Spectroscopy (FT-IR)

Functional group analysis was performed using a Shimadzu FT-IR spectrophotometer (model IR Prestige-2, SHIMADZU, Nakagyo-ku, Kyoto, Japan) equipped with an Attenuated Total Reflectance (ATR) accessory. Film samples were placed directly on the ATR crystal at room temperature (25 °C), and spectra were recorded in transmittance mode over the range of 600 to 4000 cm^−1^, with a resolution of 4 cm^−1^ and 40 scans per sample, following ASTM D5477-11 [26]. The spectra were automatically baseline-corrected prior to analysis. Fourier self-deconvolution was conducted using IR Solutions software version 2 (SHIMADZU, Nakagyo-ku, Kyoto, Japan), and peak fitting was performed using OriginPro software (version 8.5; OriginLab Corporation, Northampton, MA, USA).

#### 2.6.10. Degradability

The degradation of the films was evaluated by monitoring their mass loss after exposure to the native soil microbiota over a 15-day period. Rectangular film samples (2 × 2 cm) were prepared, weighed, and buried at a depth of 15 cm in plastic containers filled with soil. At predetermined intervals (7 and 15 days), the samples were carefully removed using tweezers, rinsed with distilled water to eliminate soil residues, and dried in an oven at 50 °C for 24 h, following the method proposed by Chandra and Rustgi [27]. The degradation percentage was determined using the following equation:

Degradability (%) = [(mi − mf)/mi] × 100(6)

where mi is the initial film mass (g), and mf is the final dry film mass (g)

### 2.7. Statistical Analysis

Data from the factorial design were analyzed using Analysis of Variance (ANOVA) at a significance level of *p* < 0.05. Regression coefficients were used to construct response surface plots using the Statistica software (version 7.0). The influence of the independent variables was ranked, and a desirability function was applied to identify the optimal formulation conditions for developing the film. Model validation was conducted by repeating the experiment under the optimal conditions predicted by desirability analysis. The experimental results were then compared with the predicted values using an independent samples *t*-test at a 95% confidence level.

## 3. Results and Discussion

### 3.1. Optimization of Film Production Conditions

Table 2 presents the experimental design matrix used to evaluate the effects of two independent variables, X_1_ (stem bark extract) and X_2_ (pod extract) of jatoba, on the total phenolic content (TPC) and antioxidant activity, assessed using DPPH and FRAP assays.

The results demonstrated that both extracts contributed to the antioxidant properties of the films, but their effects varied depending on the proportion used. Among the formulations, F8 (X_1_ = 0.50; X_2_ = 1.00) exhibited the highest TPC (16.51 mg GAE/g), along with the strongest antioxidant activity, as indicated by DPPH (76.74%) and FRAP (7.19 mmol FeSO_4_/g). This suggests a synergistic effect between the two extracts when used in combination, particularly at higher concentrations of the pod extract (X_2_).

Furthermore, it is essential to consider the different mechanisms of action of antioxidant assays. The DPPH assay evaluates free radical-scavenging capacity through hydrogen donation, and is effective in detecting antioxidants that stabilize radicals by hydrogen transfer, a typical behavior of phenolic compounds [28]. The high DPPH values, especially in formulation F8, indicate the significant presence of compounds with a high affinity for this mechanism, corroborating the high concentration of TPC in this formulation. In contrast, the FRAP assay measures the reducing power of antioxidant compounds based on electron transfer under acidic conditions [29]. The activity observed in FRAP, although lower than that in DPPH, was still significant in F8, suggesting that some of the phenolic compounds present also act through electron transfer mechanisms, albeit to a lesser extent. This contrast between the assays reinforces the complexity of the extracts, which contain compounds with different modes of antioxidative action.

In contrast, F1 (X_1_ = 0.15; X_2_ = 0.15) showed the lowest values for all response variables, indicating that low concentrations of both extracts resulted in minimal bioactive compound incorporation and antioxidant capacity. When comparing formulations with similar concentrations of one extract but varying the other (F2 and F3), it was evident that X_2_ had a greater individual impact on TPC and antioxidant activity than X_1_. The central point formulations F9–F11 (X_1_ = X_2_ = 0.50) yielded consistent and high antioxidant values, with minor variations between replicates, confirming the reproducibility and robustness of the experimental model.

Overall, the data indicated that increasing the proportion of X_2_ enhanced the phenolic content and antioxidant potential of the films, either alone or in combination with X_1_. The central and combined formulations (especially F8 and F9–F11) demonstrated optimal bioactive properties, supporting their potential for developing functional active packaging.

**Figure 1:**
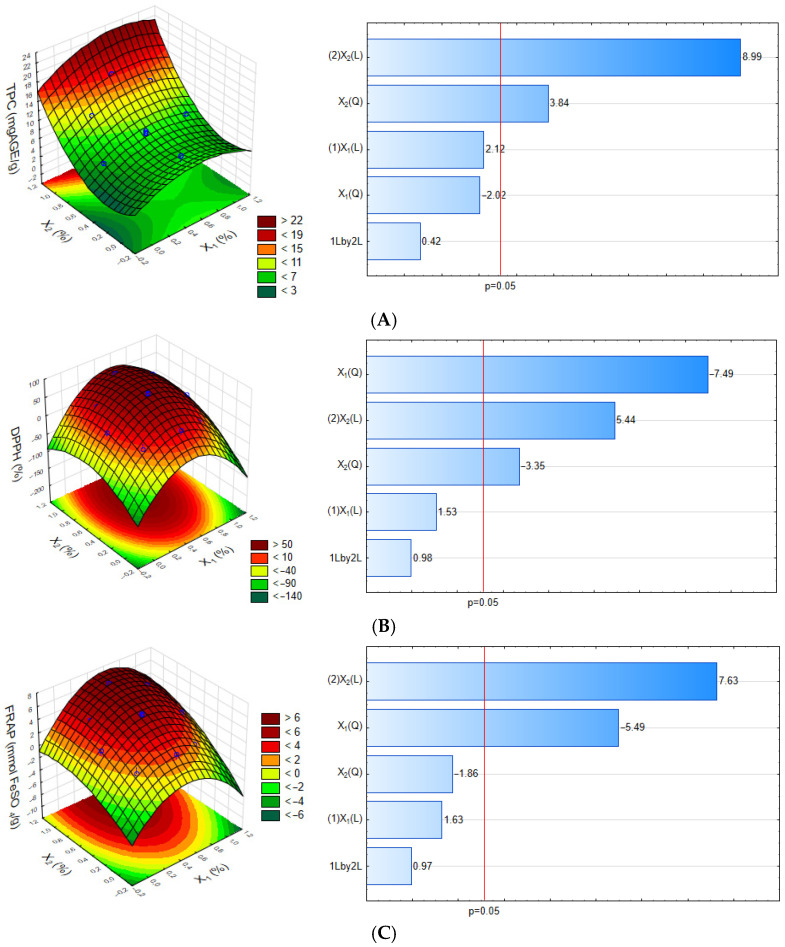
Response surface and Pareto charts related to (**A**) total phenolic content (TPC), (**B**) DPPH assay, and (**C**) FRAP assay. Note: Independent variables X1 (stem bark extract) and X2 (pod extract) of jatoba. Pareto chart and response surface plot showing the effects of the formulation variables on the measured response. X_1_(L) and X_2_(L) indicate linear effects, and X_1_(Q) and X_2_(Q) indicate quadratic effects of the stem bark and pod extracts of jatoba, respectively. 1Lby2L represents their linear interaction. Effects beyond the red line are statistically significant (*p* < 0.05).

The analysis of the Pareto chart (Figure 1A), constructed based on the experimental data obtained during the optimization process, revealed the significant effects (*p* < 0.05) of the factors on the response of each dependent variable. Among the evaluated factors, the concentration of jatoba pod extract significantly affected the total phenolic content in both linear and quadratic terms. An increase in the extract concentration resulted in films with higher levels of phenolic compounds, demonstrating a positive relationship between the extract concentration and the responses analyzed. The extraction of phenolic compounds significantly increases their concentration using different methods. The choice of extraction method and specific plant parts used play crucial roles in maximizing phenolic yields [30].

Significant effects on antioxidant activity were measured using the DPPH assay (Figure 1B). This was attributed to the quadratic concentration of the pod and stem extracts of jatoba, both of which showed a negative effect. The linear concentration of the pod extract had a positive effect. These results indicate that optimizing antioxidant activity requires precise adjustment of extract concentrations, with lower levels being essential for maximizing the antioxidant functionality of the films. Jatoba exhibits significant antioxidant properties, particularly in the seeds, which contain higher levels of phenolic compounds and exhibit greater antioxidant potential than the stems [31]. Alves-Silva et al. [9] observed the highest antioxidant activity in jatoba pulp films using the ABTS^+^ method. This finding confirms the antioxidant activity of the films containing extracts in terms of their free radical scavenging mechanism, reinforcing their potential as antioxidant packaging materials.

The linear concentration of the pod extract exhibited the strongest positive effect on the FRAP response (Figure 1C), indicating that increasing the amount of extract enhanced the antioxidant activity of the film. In contrast, the linear concentration of X_1_ had a negative effect on the same response, suggesting that reducing the concentration of the X_1_ extract increases antioxidant activity. This suggests that certain phenolic compounds may be more effective at lower concentrations, potentially due to reduced competition among the active compounds [9].

The results of this study demonstrated that the linear concentration of the X1 extract and the interaction between the independent variables X_1_ and X_2_ did not exhibit significant effects on the TPC, DPPH, and FRAP properties (*p* > 0.05). The quadratic concentration of the X_2_ extract also did not significantly influence the TPC and FRAP properties, considering a 95% confidence interval.

The response surface analysis for total phenolic content (TPC) (Figure 1A), generated using Equation 7, confirms that the concentrations of X_1_ and X_2_ exert a significant influence on TPC. A simultaneous increase in the concentrations of these independent variables resulted in a higher TPC response.

Based on Equations (7)–(9) and the response surfaces for DPPH and FRAP, the concentration of X_2_ had a significant impact, indicating that its adjustment is essential to maximize antioxidant activity. In contrast, the concentration of X_1_ showed a lesser influence on these parameters, but the analysis suggested that the optimal concentration lies around intermediate values, enabling a balance between the positive and negative effects on antioxidant performance.

TPC = 4.660 + 5.253X_2_ + 13.969X_2_^2^ + 0.000 (7)

DPPH = −39.621 − 249.85X_1_^2^ + 145.92X_2_ − 111.93X_2_^2^ + 0.000 (8)

FRAP = −0.95 − 12.40X_1_^2^ + 7.98X_2_ + 0.000 (9)

**Figure 2:**
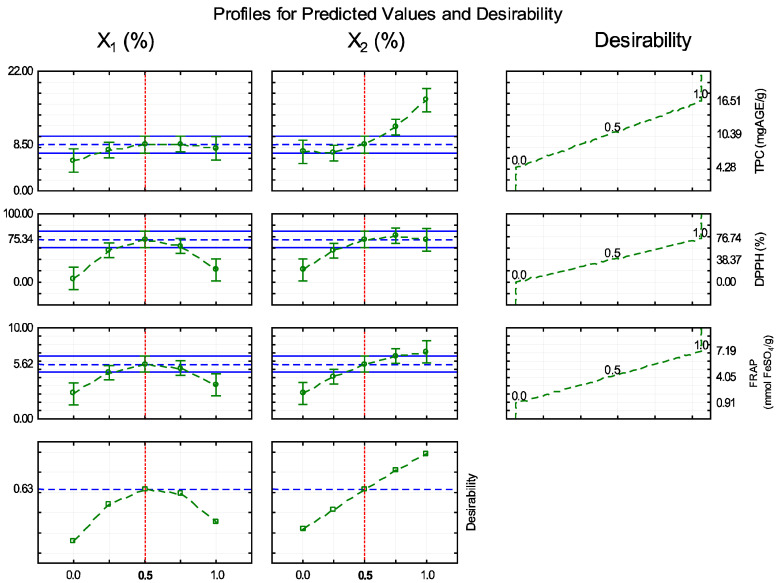
Profile plot of the predicted value and desirability. Note: Independent variables X_1_ (stem bark extract) and X_2_ (pod extract) of jatoba. The total phenolic content (TPC) and antioxidant activity were assessed using DPPH and FRAP assays.

The application of the desirability function (Figure 2) revealed that the region containing trials 9, 10, and 11, within the range of 0.5% X_1_ and 0.5% X_2_, corresponded to the optimized variables indicated by the response surface. The central point exhibited the best quality characteristics and was considered the formulation that most effectively presented the optimal conditions for obtaining an antioxidant-active film. The desirability values ranged from 0 to 1.

The central point was selected based on the maximization of the total phenolic content and antioxidant activity measured using the FRAP and DPPH methods, resulting in a film with optimized properties. The total phenolic content was 8.80 mg GAE/g, and the antioxidant activity was 75.28% by the DPPH method and 6.02 mmol FeSO_4_/g by the FRAP method, confirming its efficiency as an active antioxidant packaging. Jatoba species are characterized by high levels of phenolic compounds, which are directly associated with their remarkable antioxidant capacities. Although extracts from these species exhibit promising antioxidant properties, their antioxidant efficacy and phenolic content can vary significantly depending on the extraction methods and conditions employed [29].

The evaluation of the release of antioxidant compounds from films incorporating plant extracts is a promising approach that may pave the way for the development of biodegradable and functional materials with potential applications in various fields, especially in the food and pharmaceutical industries.

### 3.2. Characteristics of the Optimized (FO) and Control (FC) Films

The results indicate significant differences in the properties of the biodegradable semolina-based films with (0.5% X_1_ and 0.5% X_2_) and without jatoba extract, highlighting the positive impact of the bioactive extract on the polymer matrix. The results are shown in Table 3.

As shown in Table 2, the addition of the plant extract reduced the thickness of the optimized film (FO) compared to that of the control film (FC). This effect is possibly due to the interactions between the bioactive compounds present in jatoba and the polymer matrix, which may alter the viscosity and cohesion properties. Similar behavior was reported by Jafarzadeh et al. [13] for semolina-based films incorporated with nanofillers (ZnO-NPs and nano-kaolin). Alves-Silva, Romani, and Martins [9] obtained film thicknesses ranging from 0.09 to 0.20 mm using jatoba pulp. Nascimento, Calado, and Carvalho [32] analyzed starch-based films derived from passion fruit mesocarp flour and reported average thickness values between 0.133 and 0.185 mm. These values found in the literature indicate that the polymer matrix developed in this study falls within the expected range for active biodegradable films produced using the casting solution technique.

Regarding the mechanical properties, the optimized film (FO) exhibited significantly higher elongation than the control film (FC), indicating greater flexibility and deformation capacity before rupture. This behavior may be associated with the interactions between the bioactive compounds from the jatoba extract and the semolina matrix, promoting increased mobility of the polymer chains. Nguyen et al. [33] explained that the incorporation of extracts can fill the voids between molecules, resulting in more malleable films. In contrast, the tensile strength (TS) of FO was lower than that of FC, which is consistent with the observed increase in elongation; more flexible films tend to exhibit lower mechanical strength in most polymers. This inverse relationship is supported by the literature, emphasizing that the addition of bioactive compounds may plasticize the polymer matrix, thereby reducing its rigidity [34]. Therefore, increased elongation may be desirable for certain applications such as flexible packaging.

The water vapor permeability (WVP) of the optimized film (FO) was higher than that of the control film (FC). In the case of the FO film, the higher water vapor permeability may be related to a more porous or less compact polymer matrix structure, facilitating water vapor diffusion. The water vapor permeability of polymer films is influenced by various factors, including porosity, hydrophilicity, and structural organization [35]. More porous or less compact structures favor vapor diffusion, regardless of the material’s solubility. Additionally, hydrophilic polymers have a greater affinity for polar molecules, such as water vapor, resulting in higher WVP values than those of more hydrophobic polymers. The polarity, homogeneity, and internal structure of the film components also significantly affect these properties [36]. Furthermore, these structural changes may be associated with the formation of microchannels or alterations in matrix compaction due to the presence of phenolic compounds [37]. Nevertheless, the values obtained are consistent with those reported in the literature for polysaccharide-based films enriched with plant extracts, indicating that the optimized film still presents suitable characteristics for food packaging applications [16].

Water solubility was high in both films (FO and FC), with no statistically significant difference. This result is expected for polysaccharide-based films, such as those made from semolina, which exhibit a high affinity for water [38]. The incorporation of jatoba extract did not significantly alter this property, suggesting that the polymer matrix retained its predominantly hydrophilic character [6]. The solubility observed in the film may be explained by the stronger intermolecular interactions within the polymer matrix. Strong hydrogen bonds between the polymers can increase the structural cohesion of the film, reducing its ability to disperse in an aqueous medium [39]. Thus, while WVP is related to the ease of passage of vapor molecules, solubility reflects the matrix’s resistance to complete disintegration and is influenced by distinct factors. However, the literature reports solubility values for semolina-based films ranging from 37.51 to 45.14% [13] and for films containing jatoba pulp from 30.6 to 35.7% [9], which are lower than those found in this study.

**Figure 3:**
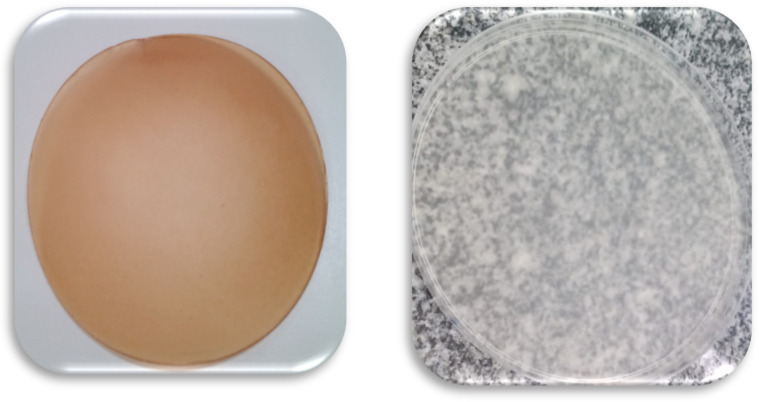
The FO (optimized film) and FC (control film) appearance.

Regarding the color properties, the addition of jatoba extracts to the semolina-based film significantly altered the colorimetric parameters (L, a, and b*), imparting a characteristic hue to the material. The optimized film FO exhibited lower lightness than the control film FC, indicating a less bright and more opaque appearance. Additionally, the a* and b* values were significantly higher in the FO than in the FC, reflecting a warmer color with predominant reddish and yellowish tones. The total color difference (ΔE) between the films was also substantial, confirming that the presence of the extract induced a perceptible change in the visual appearance of the material, as shown in Figure 3.

This amber-tinged color profile is characteristic of materials containing phenolic compounds and other secondary metabolites present in plant extracts, such as flavonoids and tannins [40]. Studies have shown that Amber coloration is common in films produced from plant extracts rich in bioactive compounds, such as those derived from tea, vegetable peels, and pods. These amber-colored materials not only provide distinct aesthetic appeal but are also associated with antioxidant functionality, as the color is often indicative of the presence of compounds capable of neutralizing free radicals [6].

In addition to its visual aspect, amber coloration in packaging materials may have important technological implications. Darker films can protect food from light exposure, thereby reducing the degradation of sensitive compounds, such as vitamins and natural pigments, and slowing lipid oxidation reactions. This functionality is particularly relevant for packaging light-sensitive products, such as oils, nuts, and wholemeal flours [41]. The use of amber-colored films is also a well-established strategy in industrial packaging, as seen in the case of amber bottles used for light-sensitive beverages (such as beer and tea), whose function is to filter UV radiation and preserve product quality [42]. Thus, the amber hue imparted by jatoba extracts to the semolina film may add functional value to the material, in addition to providing visual differentiation in the sustainable packaging market [9].

Therefore, the results obtained in this study demonstrate that amber coloration is not merely an aesthetic feature but also an indicator of the presence of bioactive compounds in the film, which may play a protective role against food oxidation, thereby expanding the material’s applications as an active packaging. Coloration also reinforces the appeal of sustainability and innovation, as it adds value to natural byproducts, such as jatoba extracts, and connects the functional benefits to the structural properties of the film.

### 3.3. Thermal Properties

**Figure 4:**
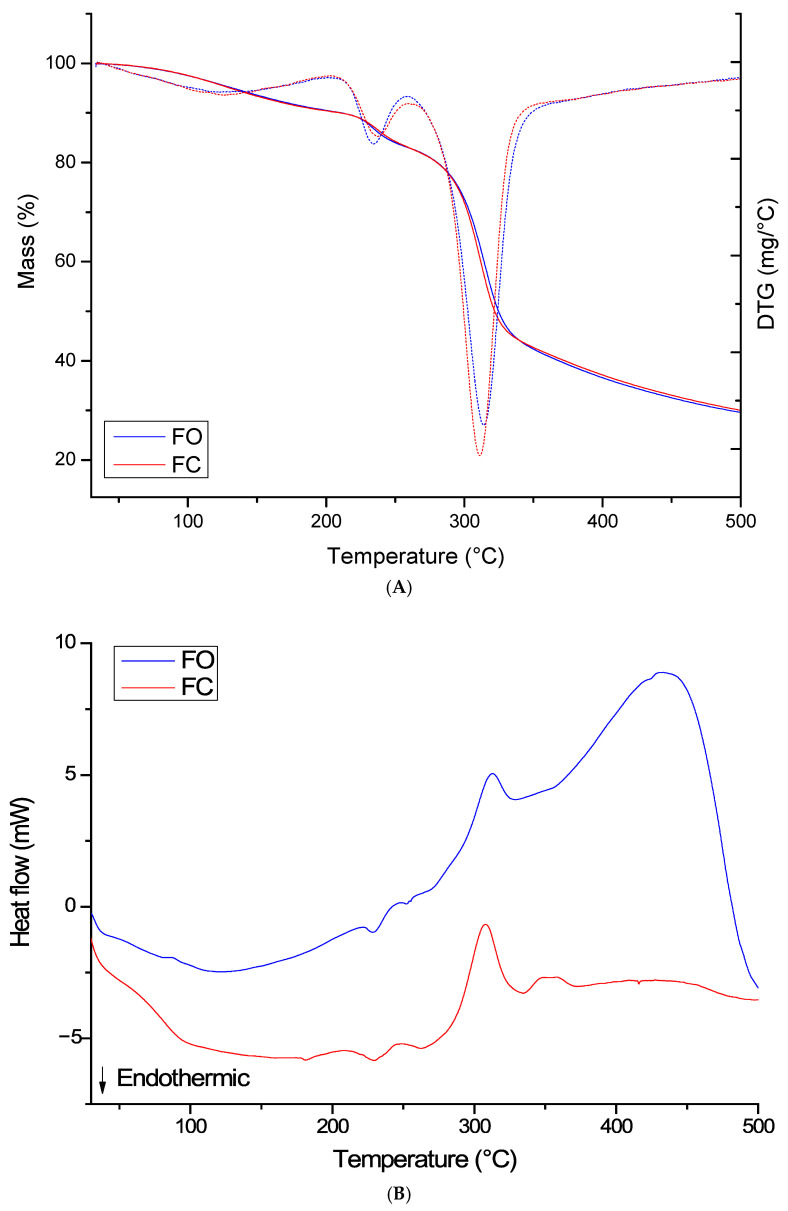
(**A**) TGA/DTG curves and (**B**) DSC curves of the FO and FC films.

Thermogravimetric analysis (Figure 4A) revealed three main degradation stages for semolina-based films, both in the optimized formulation with jatoba extracts (FO) and in the control formulation (FC). These stages are associated with distinct thermal processes characteristic of polymeric materials of organic origin.

In the first stage, corresponding to the range of 59–170 °C for FO and 69–197 °C for FC, the mass loss was associated with the evaporation of free water and the release of low-molecular-weight volatile compounds, including thermally unstable fractions present in the plant extract. The initial degradation temperature (Tonset) was slightly lower for FO (59 °C), indicating that the jatoba extracts contributed to greater initial thermal sensitivity, possibly due to the presence of phenolic and naturally unstable volatile compounds [43].

The second stage, occurring between 212 and 255 °C for OF and 214 and 260 °C for FC, reflects the thermal degradation of the main constituents of the polymer matrix, especially semolina polysaccharides [13]. The maximum degradation temperatures (Tmax) were similar between the films (235 °C for FO and 237 °C for FC), indicating that the addition of jatoba extracts did not significantly alter the thermal stability of the matrix at this stage. The mass loss in this stage was approximately 7% for both formulations, a result consistent with that of biopolymeric films containing natural additives [38].

In the third stage, beginning around 273 °C and concluding near 350 °C, degradation of more recalcitrant components of the matrix was observed, including longer polysaccharide chains, cross-linkages, and Maillard reaction products [44]. The Tmax was slightly higher for FO (313 °C) than for FC (311 °C), suggesting a possible protective effect of the jatoba extracts, potentially related to the antioxidant activity of phenolic compounds, which may delay oxidative degradation at elevated temperatures. The final residual mass (24% for FO and 23% for FC) indicates that both films maintained good thermal stability up to approximately 350 °C, which is favorable for packaging applications, particularly those requiring a certain level of thermal resistance during processing or storage [6].

In comparison, films based on synthetic polymers, such as polyethylene or polystyrene, exhibit degradation profiles with only one or two well-defined stages and decomposition temperatures generally above 400 °C, but with lower biodegradability and higher residual toxicity. In contrast, biodegradable films made from starch, gelatin, pectin, or carboxymethylcellulose also display multiple degradation stages, with Tmax values ranging from 220 °C to 320 °C, similar to those observed in this study [45]. Thus, the films containing jatoba extract exhibited thermal behavior consistent with that of established biopolymers, indicating their technical feasibility for similar applications.

DSC analysis (Figure 4B) confirmed the thermal events observed in the TGA, providing additional information on the endothermic and exothermic transitions of the sample. In the FO, endothermic peaks were observed at 120 °C, 230 °C, and 251 °C, corresponding to the loss of free water, molecular rearrangement reactions, and possible partial melting of the polymer matrix, respectively [46]. The exothermic events at 315 °C and 430 °C are associated with the degradation of macromolecules and the combustion of organic residues. The presence of multiple endothermic and exothermic peaks highlights the heterogeneous nature of the system, composed of semolina and jatoba extracts, containing different types of bonds and thermally active components [9].

For the control film (FC), endothermic peaks were observed at 150 °C, 230 °C, and 260 °C, while exothermic peaks were observed at 307 °C and 355 °C. The slightly higher thermal transition values in the FC may indicate greater rigidity of the polymer matrix in the absence of the plant extract, which acts as a natural plasticizer, promoting increased molecular chain mobility [34].

The difference in the thermal behavior of the films can also be interpreted in light of the interactions between the phenolic compounds in the jatoba extracts and the functional groups of semolina starch. Studies have indicated that such interactions can modify the melting enthalpy, enhance the heat absorption capacity, and alter the thermal stability of biodegradable polymeric systems [42]. Compared to biopolymers such as chitosan or whey protein, the films in this study exhibited comparable thermal stability, with the added advantage of bioactive compounds that confer antioxidant properties [43].

### 3.4. Structural Characterization by X-Ray Diffraction (XRD)

**Figure 5:**
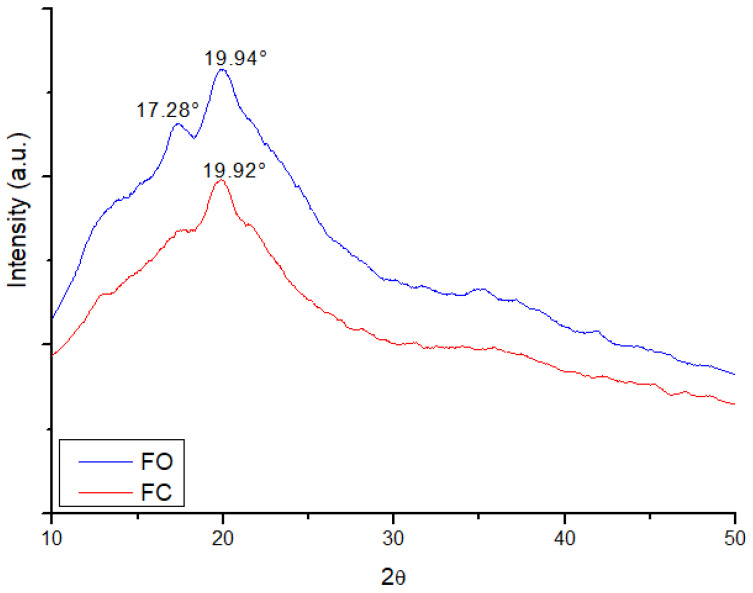
XRD curves of the films (FO and FC).

X-ray diffraction (XRD) (Figure 5) was used to evaluate the crystallinity and atomic arrangement of the films. X-ray diffraction (XRD) analysis revealed significant structural differences between the control films (FC) and the optimized films containing jatoba extracts (FO), demonstrating the impact of the addition of bioactive compounds on the crystalline organization of the semolina-based polymer matrix.

In the FC spectrum, two diffraction peaks were identified, with the main peak at 17.28°, corresponding to a type-B crystalline structure. This pattern is typical of retrograded starches, whose formation is associated with the reorganization of starch chains during the cooling and drying of the film, particularly due to the realignment of short amylopectin chains into double helices [47]. The presence of this peak suggests a semi-crystalline structure, which is characteristic of polymeric films composed predominantly of starch, reinforcing the influence of the intrinsic polymer structure on the spatial organization of the material [45].

In contrast, the XRD pattern of FO shows a broad peak at 19.94°, associated with a predominantly amorphous pattern. This result indicates that the incorporation of jatoba extracts reduced the crystallinity of the semolina matrix, possibly due to chemical interactions between bioactive compounds (such as phenols and flavonoids) and the polymer chains of the starch. This behavior is consistent with the findings of Lipatova, Yusova, and Makarova [48], who observed that the inclusion of natural additives in polymeric matrices can hinder the ordered organization of chains, reduce the formation of crystalline zones, and promote structural disorder.

Previous studies have corroborated these observations. Rodrigues et al. [49] identified a predominantly amorphous pattern in chitosan/gelatin films containing jatoba resin, reinforcing the tendency of plant extracts rich in phenolic compounds to interfere with the crystallinity of the polymer matrix. Similarly, Biswas et al. [45] reported peaks at 19.95° in enzymatically treated semolina starch, associating this reflection with a type B structure, while peaks around 17° were attributed to the organization of amylose and amylopectin in more ordered regions.

It is important to highlight that Roy and Rim [50] also associated the presence of broad and diffuse peaks, such as those found at 19.92° in the FC and 19.94° in the FO, with the amorphous nature of plant-derived polymers, such as pectin, which is frequently used in biodegradable films. The authors emphasized that broader peaks reflect the predominance of amorphous regions, while narrower and more intense peaks indicate crystalline zones. Thus, the peak at 17.28° in the FC reinforces the presence of semi-crystalline structures associated with the starch matrix, whereas the broader profile at 19.94° in the FO suggests greater molecular disorder, likely resulting from interactions between the compounds in the jatoba extracts and the semolina matrix.

These results indicate that the addition of jatoba extracts, although promoting a slight increase in structural disorder, contributed to the formation of a more heterogeneous and functional polymeric system with potential applications in active packaging products. The reduction in crystallinity, often associated with greater flexibility and capacity to absorb bioactive compounds, may be beneficial for the antioxidant performance and functionality of the material [34].

### 3.5. FTIR Spectrophotometry of the Films

**Figure 6:**
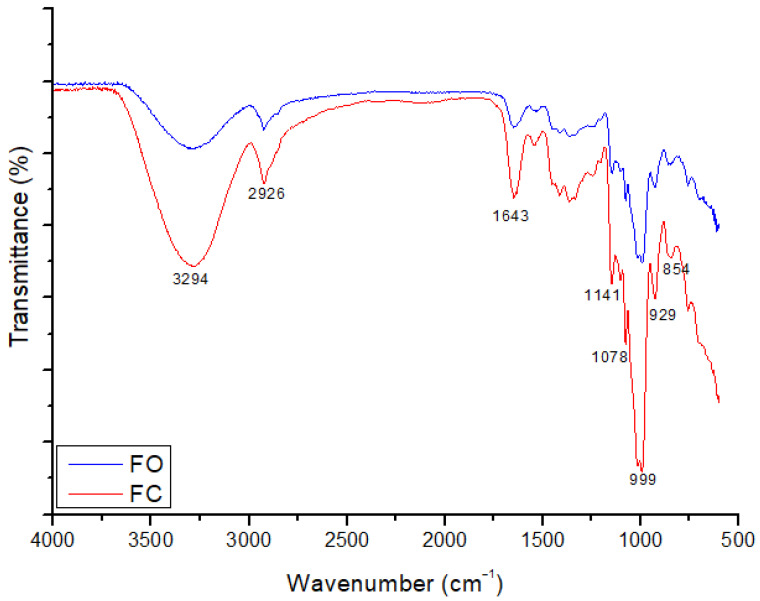
FTIR spectra of the films (FO and FC).

FTIR spectroscopy was used to identify and determine the functional groups of the compounds present in the films. FTIR spectra of films are shown in Figure 6.

The obtained spectrum exhibited bands in the region of 3296 to 3314 cm^−1^, attributed to the stretching vibration of the hydroxyl group (–OH), indicating the presence of hydrophilic functional groups derived from both the polymer matrix and the incorporated additives [51]. Bands between 2916 and 2930 cm^−1^ are associated with the stretching of C–H bonds, which are common in organic chains [52]. The additives used in the formulations contain phenols, which feature active hydrophilic functional groups such as hydroxyl, ether, and carbonyl groups. The presence of O–H, C–O, and C–O–C functional groups in both the additives and polymer matrix likely contributed to enhanced intermolecular interactions, predominantly through hydrogen bonding. These interactions are considered key factors in modulating the mechanical behavior of the films, particularly in terms of their flexibility and strength [53]. The structural complexity and concentration of jatoba phenolics may have increased their binding affinity with starch molecules, interfering with polymer aggregation and increasing matrix disorder, which helps explain the observed changes in the mechanical performance of the material [54].

Bands located in the range of 1337 to 1412 cm^−1^ are attributed to the angular deformation of C–H bonds, which is typical of polysaccharide structures [55]. The bands at 1141 cm^−1^ and between 1074 and 1078 cm^−1^ correspond to the stretching of C–O and C–O–C bonds, related to glycosidic linkages and the cyclic structures present in polysaccharides [56].

The bands observed at 999 cm^−1^ and 929 cm^−1^ were associated with the angular deformation vibrations of the C–O group, which are characteristic of carbohydrate structures such as fructose and glucose, which are abundantly present in the film formulation. These bands may also reflect molecular vibrations associated with the presence of plasticizers or specific interactions between the plasticizers and other components of the polymer matrix [57]. Specifically, the band at 929 cm^−1^ is related to the presence of plasticizers added to the films [58]. This region is considered a fingerprint zone used to differentiate molecular compounds, a distinction that is clearly evidenced in the obtained spectrum [59].

### 3.6. Soil Degradation

The simulated soil degradation analysis revealed significant differences in the behavior of the FC and FO films, highlighting the impact of bioactive compound addition on the degradation rate. The FO film showed a marked mass loss, with degradation starting as early as the seventh day, reaching rates of 72.95% and 83.47% by the fifteenth day. In contrast, the FC film began to degrade only on the fifteenth day, with a degradation rate of 9.3%, indicating a significantly slower breakdown.

The difference in the behavior of the films can be attributed to the presence of jatoba extract in the FO formulation. Bioactive compounds found in jatoba, such as phenols, tannins, and lignin, are known to act as additional sources of carbon and nutrients for soil microorganisms [49]. The higher availability of these compounds in the FO film likely stimulated microbial activity and accelerated the degradation process. Furthermore, the structural modification of the film due to the presence of the extract may have increased its porosity and surface roughness, facilitating the penetration of water and microbial enzymes, which are critical factors for biodegradation in natural environments [56].

In contrast, the control film (FC), although biodegradable over a longer time frame, exhibited a more compact and less porous structure. The absence of additional bioactive compounds may have limited the supply of readily assimilable nutrients, reducing microbial colonization and activity on the film surface, which explains the slower degradation rate [48].

The rapid degradation rate of FO (over 80% in 15 days) represents a considerable advantage in applications where accelerated biodegradation is desirable, such as in disposable packaging, agricultural coatings, and single-use products. This behavior aligns with the current demand for more sustainable and environmentally responsible solutions. Reduced environmental residence time helps mitigate the negative impacts associated with plastic waste accumulation, which is one of the greatest global challenges in terms of sustainability [2].

Finally, although FC exhibited a slower degradation rate, its composition, which is predominantly based on semolina, a natural polysaccharide, ensures that it is also biodegradable over time. This reinforces the potential of both materials as more sustainable alternatives to petroleum-based synthetic polymers, such as polyethylene and polystyrene, which can take hundreds of years to degrade.

## 4. Conclusions

The present study successfully developed biodegradable films based on semolina flour starch, enriched with hydroalcoholic extracts from the stem bark and pod of jatoba, combined with passion fruit pectin, sucrose, and inverted sugar as plasticizers. The results revealed significant improvements in the physical and functional properties of the films, including greater elongation, higher phenolic compound content, and notable antioxidant activity. Additionally, the changes in color parameters imparted a characteristic brick-red hue to the material. The optimized films, produced with 0.5% extracts from the stem bark and pod of jatoba, exhibited satisfactory thermal stability, low solubility, and an increase in water vapor permeability, making them promising materials for use in active antioxidant packaging, particularly for food products susceptible to oxidation. Moreover, degradation tests indicated that the optimized film has an accelerated decomposition rate in soil, representing an advantage for single-use and disposable packaging applications.

Future studies should explore the application of the developed films in real food packaging systems, assessing their performance in direct contact with different types of products and under various storage conditions. Complementary investigations on the controlled release of bioactive compounds, interactions with specific food matrices, and sensory evaluation of packaged products may enhance the understanding of the practical functionality of the developed films. Furthermore, evaluating the antimicrobial activity of the films and incorporating other natural extracts or bioactive agents may represent promising strategies to enhance the functional activity of packaging and diversify its commercial applications within the context of sustainability and technological innovation.

## Figures and Tables

**Table 1:** Levels of independent variables X_1_ (stem bark extract) and X_2_ (pod extract) of jatoba.

Independent Variables	Levels				
	−α	−1	0	+1	+α
X_1_ (%)	0.00	0.15	0.50	0.85	1.00
X_2_ (%)	0.00	0.15	0.50	0.85	1.00

**Table 2:** Design matrix: Independent variables X_1_ (stem bark extract) and X_2_ (pod extract) of Jatoba. The dependent variables were total phenolic content (TPC) and antioxidant activity (DPPH and FRAP).

Independent Variables			Response Variables		
	X_1_ (%)	X_2_ (%)	TPC (mgGAE/g)	DPPH (%)	FRAP (mmol FeSO_4_/g)
F1	0.15	0.15	4.28	0.00	0.91
F2	0.15	0.85	11.68	34.05	4.28
F3	0.85	0.15	5.28	5.02	1.43
F4	0.85	0.85	13.57	58.12	6.08
F5	0.00	0.50	6.08	16.25	2.75
F6	1.00	0.50	8.53	25.48	3.26
F7	0.50	0.00	8.55	32.57	2.83
F8	0.50	1.00	16.51	76.74	7.19
F9	0.50	0.50	8.80	74.89	5.73
F10	0.50	0.50	8.25	75.28	5.52
F11	0.50	0.50	8.45	75.87	5.63

**Table 3:** Physical, chemical, and structural properties of the optimized film (FO) and control film without jatoba extract (FC).

Parameters	FO	FC
Thickness (mm)	0.150 ± 0.02 b	0.160 ± 0.02 a
Elongation at break (%)	23.82 ± 4.71 a	8.22 ± 0.53 b
TS (MPa)	5.62 ± 1.77 b	7.39 ± 1.73 a
WVP (10^−4^ gH_2_O·mm/m^2^·h·mmHg)	1.84 ± 0.10 a	1.31 ± 0.11 b
Water solubility (%)	65.40 ± 9.90 a	63.00 ± 1.80 a
L*	49.93 ± 1.30 b	59.77 ± 0.88 a
a*	4.24 ± 0.72 a	−2.02 ± 0.15 b
b*	20.16 ± 1.36 a	1.23 ± 0.30 b
ΔE	22.23	-

Note: Values are presented as mean ± standard deviation (mean ± SD). Different superscript letters (a, b) in the same row indicate significant differences (*p* < 0.05, *t*-test). The optimized film (FO) contained 0.5% X_1_ and 0.5% X_2_, and the control film (FC) did not contain jatoba extracts. Water Vapor Permeability (WVP) and Tensile Strength (TS).

## Data Availability

The original contributions presented in the study are included in the article. Further inquiries can be directed to the corresponding author.
